# Does maternal age or related factors influence the appearance of psychopathology in children?

**DOI:** 10.1192/j.eurpsy.2021.400

**Published:** 2021-08-13

**Authors:** J. Buesa Lorenzo, A. García-Blanco, A. Moreno-Giménez, L. Campos Berga, R. Sahuquillo-Leal, A. Nowak, D. Hervás, V. Diago, M. Vento

**Affiliations:** 1 Psychiatry, University and Polytechnic Hospital La Fe, Valencia, Spain; 2 Neonatal Research Group, The Medical Research Institute Hospital La Fe (IIS La Fe), Valencia, Spain; 3 Psychiatry, Adam Mickiewicz University, Poznań, Poland; 4 Data Science Unit, Biostatistics, And Bioinformatics, The Medical Research Institute Hospital La Fe (IIS La Fe), Valencia, Spain; 5 Gynaecology And Obstetrics, University and Polytechnic Hospital La Fe, Valencia, Spain

**Keywords:** predictor, PSYCHOPATOLOGY, maternal age

## Abstract

**Introduction:**

Maternal age and related factors, such as social vulnerability, are associated with neurodevelopmental and behavioral disorders in offspring.

**Objectives:**

To examine the influence of maternal age and its related factors on the appearance of autism spectrum disorder (ASD), attention deficit hyperactivity disorder (ADHD), alterations in executive functions and behavioral syndromes of the offspring.

**Methods:**

A prospective study was conducted, consisting of 131 healthy pregnant women aged 20 to 41 years, recruited at 38 weeks’ gestation. Their offspring were followed up to 2 years after birth, when psychopatology was assessed. Maternal age and possible related factors were considered predictors. Bayesian ordinal regression models were performed for each outcome variable.

**Results:**

Symptoms of ASD in children were associated with an older maternal age (OR = 0.188; 95% CI[1.062, 1.401]) and a lower educational level of the parents (OR = -0.879; 95% CI[0.202, 0.832]), meanwhile poor social support predicted most ADHD symptoms OR = -0.086; 95% CI[0.838, 1]) and executive dysfunctions OR = -0.661; 95% CI[0.313, 0.845]. Lower parental education predicted both externalizing and internalizing behavior.

**Conclusions:**

Maternal age-related factors were the main predictors of neurodevelopmental disorders in offspring, rather than maternal age. The performance of prenatal interventions in pregnant women with advanced age and anxious depressive symptoms or adverse social situation, is crucial to reduce the risk of neurodevelopmental disorders in the offspring. Likewise, being able to carry out an early detection of childhood psychopathology would allow the implementation of resources that improve their long-term prognosis.

**Disclosure:**

No significant relationships.

